# High‐Shear Enhancement of Biginelli Reactions in Macromolecular Viscous Media

**DOI:** 10.1002/marc.202400490

**Published:** 2024-09-25

**Authors:** Aaron Hung Bui, Naomi Beth Rowlands, Anne Dilpashani Fernando Pulle, Sam Andrés Gibbs Medina, Tullia Jade Rohrsheim, Bryan Tyler Tuten

**Affiliations:** ^1^ School of Chemistry and Physics Centre for Materials Science Queensland University of Technology (QUT) 2 George Street Brisbane QLD 4000 Australia; ^2^ Department of Chemistry and Biochemistry University of Texas at Tyler 3900 University Boulevard Tyler Texas 75799 USA

**Keywords:** biginelli reaction, multicomponent reaction, post‐polymerization modification, vortex fluidic device

## Abstract

Chemical reactions and transformations in non‐traditional vessels have gained significant interest in recent years. Flow chemistry, with its advantages in mixing, mass transfer, scalability, and automation, is a driving force behind this paradigm shift. In particular, the Vortex Fluidic Device (VFD) has emerged as a versatile tool across various applications, from organic synthesis to materials science. In this study, the role of the VFD in performing the Biginelli reaction, a multicomponent reaction widely used in pharmaceutical and polymer science, for a post‐polymerization modification is explored. By conducting the Biginelli reaction in the VFD, rapid product formation with low catalyst loading and without the need for high temperatures is achieved. However, the critical need to understand and know solution viscosity, especially within the context of modifying macromolecules is highlighted.

## Graphical abstract

Biginelli reactions are important multicomponent reactions in macromolecular chemistry. it is demonstrated that by using the vortex fluidic device (VFD) Biginelli reactions can be completed at room temperature in just 1 h. However, the critical need to understand solution viscosity as a function of macromolecular chain length to better understand polymer modifications within the VFD is highlighted.

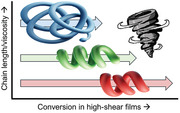

## 1 Introduction

Chemical reactions and transformations in “non‐traditional” reactors have seen an explosion of interest in recent years. The movement toward exploring alternative reactor designs beyond the standard round bottom flask has largely been driven by the vast work within the flow chemistry community. Flow chemistry has numerous advantages over standard flask‐based chemistries such as high rates of mixing, excellent mass and heat transfer, scalability, and automation.^[^ ^1^, ^2^ ^]^. Just as in the broader chemical community, flow chemistry has found numerous applications within macromolecular chemistry including enhanced rates of polymerization,^[^ ^3^, ^4^ ^]^ post‐polymerization,^[^ ^5^ ^]^ production of sequence‐defined polymers,^[^ ^6^ ^]^ and up‐scaled synthesis of single‐chain polymer nanoparticles.^[^ ^7^ ^]^ While obviously a powerful tool in macromolecular chemistry, flow chemistry still has some drawbacks in this space, predominantly from highly viscous, high molecular weight macromolecules becoming more difficult to pump and have efficient mixing in narrow‐diameter flow chemistry tubing. Various methods to overcome this challenge within the “non‐traditional” reactor space include utilizing a ball mill,^[^ ^8^, ^9^, ^10^, ^11^ ^]^ and pseudo‐2D flow reactors such as spinning disc reactors,^[^ ^12^, ^13^, ^14^ ^]^ and more recently the Vortex Fluidic Device (VFD).^[^ ^15^ ^]^

First introduced in 2012, the VFD has gained utility across a wide range of applications including organic synthesis,^[^ ^16^, ^17^, ^18^, ^19^, ^20^, ^21^, ^22^ ^]^ food science,^[^ ^23^, ^24^ ^]^ medicinal chemistry,^[^ ^25^ ^]^ and materials science.^[^ ^26^, ^27^ ^]^ Recently, our team established that the high‐shear mixing in the VFD is an excellent tool for carrying out “four” component Passerini polymerizations of bulky monomers, something that cannot be done in traditional flask chemistry at standard conditions.^[^ ^15^ ^]^ Thus, we set out to explore the role of macromolecules and viscous media play on the enhancement of another highly useful multicomponent reaction, the Biginelli reaction.

Developed by Pietro Biginelli, the Biginelli reaction requires an aromatic aldehyde, urea (or thiourea), and a beta keto ester which combine to form 3,4‐dihydropyrimidin‐2(1H)‐ones. Used widely across many pharmaceutical applications such as anti‐fungal,^[^ ^28^ ^]^ anti‐cancer,^[^ ^29^ ^]^ and pesticides,^[^ ^30^ ^]^ the Biginelli reaction has also found use in polymer science via the synthesis of materials with tunable high glass transition temperatures,^[^ ^31^, ^32^ ^]^ polymers with anticancer properties,^[^ ^33^ ^]^ monomer synthesis for UV‐protective polymers,^[^ ^34^ ^]^ and adhesives.^[^ ^35^ ^]^ While highly atom‐economical and fairly straightforward to carry out, the Biginelli reaction still faces some drawbacks by way of requiring catalysts, high temperatures, and long reaction times.^[^ ^36^ ^]^ Herein, we introduce the VFD as a means to rapidly enhance the reaction speed, maintain low catalyst loadings, and operate at ambient temperatures the Biginelli reaction. Furthermore, we highlight the scope of the VFD to perform Biginelli‐based post‐polymerization modifications (PPMs) on increasing molecular weight poly(ethylene glycol) (PEG) chains, and therefore, increasingly high solution viscosities.

## 2 Results and Discussion

**Scheme 1:**
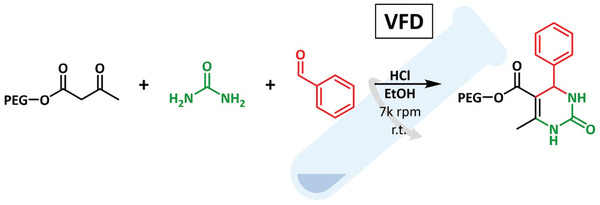
Biginelli reaction in the Vortex Fluidic Device (VFD).

In order to elucidate the feasibility and ultimately the appropriate reaction conditions for high‐shear enhanced Biginelli reactions, we first explored a simple Biginelli reaction in the VFD using traditional starting materials (**BA0**): ethyl acetoacetate, urea, and benzaldehyde in ethanol catalyzed by 20 µL (one drop) of concentrated hydrochloric acid (**Scheme** **1**).^[^ ^37^ ^]^ Typically, Biginelli reactions require high temperatures (reflux) as well as reaction times of up to 24 h.^[^ ^36^, ^38^ ^]^ Thus, we set out to identify if both temperature and reaction time for Biginelli reactions could be reduced via the use of high‐shear mixing found in the VFD. All reactions were carried out in batch mode within a sealed VFD tube so that reagent concentration could be controlled (i.e. minimize evaporation), thus leaving only solution viscosity as the primary variable. When running the VFD in continuous flow mode, the end of the tube cannot be sealed, thus without a detailed understanding of solvent evaporation rates within a flowing VFD, chemical transformations at the end of the reaction will generally be exposed to higher viscosities (due to evaporation) than reactions at the beginning of the reaction. Building off our previous work in high‐shear enhanced multicomponent reactions, we chose 7000 rpms and a total volume of 0.5 mL.^[^ ^15^ ^]^ Analysis of the small molecule Biginelli reaction confirmed that Biginelli reactions could be pushed to high yield at ambient temperature and in only 60 min. This is confirmed via ^1^H nuclear magnetic resonance (NMR) spectroscopy showing full conversion of the starting materials to the Biginelli product (Figure S1, Supporting Information), whose mass is further confirmed by mass spectrometry (MS).

Encouraged by the promising results of the VFD as a powerful tool in enhancing the Biginelli reaction, we set out to probe the effects of solution viscosity on Biginelli‐based post polymerization modifications on increasingly larger PEG chains. We selected various commercially available poly(ethylene glycol) monomethyl ethers ranging from Mn 350 to Mn 10 000 g mol^−1^ and 2‐methoxyethanol to functionalize the free hydroxyl side with *tert*‐butyl acetoacetate. Catalyzed by 20 µL (one drop) of concentrated hydrochloric acid, our acetoacetylated molecules along with urea and benzaldehyde formed the Biginelli adduct.

2‐Methoxyethanol (a “pseudo‐PEG” moiety) (**AA1** and **BA1**), acting as the small molecule analog to the poly(ethylene glycol), served as our initial test demonstrating the effectiveness of the VFD to yield a pure Biginelli product rapidly (60 min), with low catalyst loading, and without the need for high temperatures (Figure S7, Supporting Information). This first example is in line with our initial exploration of the small molecule test reaction **BA0**.

**Figure 1:**
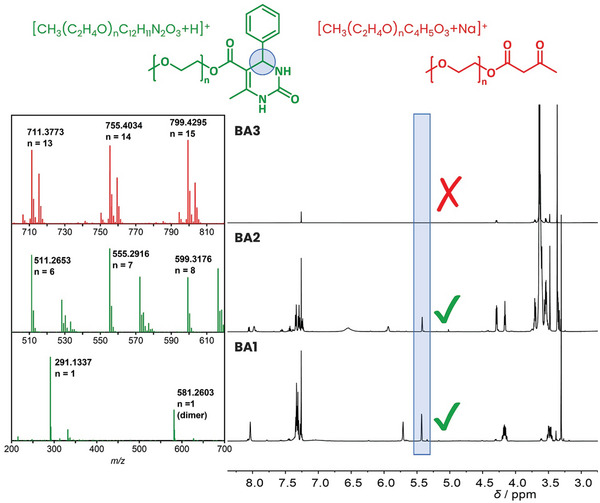
Stacked NMR and MS spectra of products after PPM reaction. Note: The mass spectrum of BA2 displays the PEG repeat unit associated with two different cations; the unlabelled one refers to NH_4_^+^ as a cation. For BA1 n ≈1, for BA2 n ≈ 8, and for BA3 n ≈ 15.

With varying molecular weights (Mn 350, 750, 1,900, 5 000, and 10 000 g mol^−1^) of PEG monomethyl ether, we began investigating the ability of the VFD to carry out Biginelli PPMs with increasing solution viscosity (as a function of PEG chain length). In the first instance, we converted the free hydroxyl functionality of our PEG monomethyl ethers into the acetoacetate functionality needed for the subsequent Biginelli modification. Proton NMR spectra and MS analysis confirmed the successful end‐group functionalization (Figures S3–S6, Supporting Information). We performed the Biginelli PPM reaction on our acetoacetylated PEG monomethyl ether Mn 350 (**AA2**) first. The measured NMR spectrum shows the emergence of a chiral proton peak (designated “f” in Figure S8, Supporting Information), associated with the formation of the Biginelli adduct (**BA2**). MS analysis also revealed the masses of PEG monomethyl ether chains of different lengths capped with the Biginelli moiety (Figure S9, Supporting Information). While both NMR and MS analysis confirmed that the Biginelli adduct was formed, they also show incomplete conversion as indicated by starting material signals remaining in the NMR spectra. We then proceeded to follow the same procedure for our PEG monomethyl ether Mn 750. The successful acetoacetylation (Figure S5, Supporting Information) was followed by the Biginelli PPM. However, going from Mn 350 to 750 g mol^−1^, resulted in a dramatic decrease in Biginelli reaction conversion. The larger polymer showed no traces of a successful Biginelli PPM as the NMR measurement confirmed (Figure S10, Supporting Information). As can be seen in **Figure** **1**, the spectra for the polymer showed no difference pre and post Biginelli reaction.

Contrasting the NMR spectra for the Biginelli reactions of AA1, AA2, and AA3 confirms this (Figure 1). No chiral proton peak can be detected in the acetoacetylated PEG monomethyl Mn 750 sample indicating that no Biginelli reaction has occurred.

Due to lack of conversion of the Biginelli PPM of PEGs above Mn 750 g mol^−1^, only experimentals for PEG monomethyl ether Mn 350 (**AA2** and **BA2**) and 750 g mol^−1^ (**AA3** and **BA3**) were included.

Knowing that polymer solutions of varying molecular weight display different viscosities and considering the complex fluid dynamics that occur in the VFD during operation,^[^ ^20^, ^21^, ^23^ ^]^ this led us to explore the role that solution viscosity plays when using the VFD as a vessel for reactions that involve materials that can significantly alter the viscosity of the reaction solution. To test our hypothesis, we prepared solutions of 2‐methoxyethanol, PEG monomethyl ether Mn 350 g mol^−1^ (PEG‐350), and Mn 750 g mol^−1^ (PEG‐750) at the same concentrations as during the original PPM reaction and measured the viscosities of these solutions.

**Figure 2:**
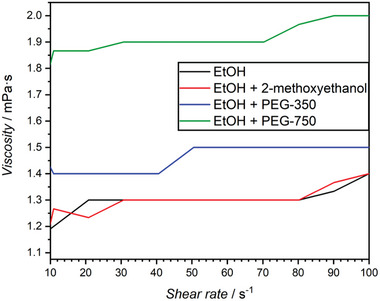
Low‐viscosity measurements of 2‐methoxyethanol, PEG‐350 and PEG‐750 in ethanol. As the Biginelli reactions were carried out in ethanol as the solvent, pure ethanol was measured for reference. Measured viscosities increase with a larger molar mass of molecules dissolved.

From the measurements (**Figure** **2**), we deduce that the addition of 2‐methoxyethanol has no significant effect on viscosity when compared to pure ethanol as a solvent. This changes when PEG‐350 and PEG‐750 are added to the solution. While PEG‐350 results in a slight increase in viscosity, PEG‐750 has a greater impact. This test highlights the critical role that solution viscosity plays on Biginelli reactions, suggesting that a solution viscosity below 1.5 mPa·s must be maintained in order to successfully complete a Biginelli reaction regardless of whether it is a small molecule reaction or a PPM. To test this upper viscosity limit, we prepared a solution identical to the PEG‐750 solution exhibiting a solution viscosity of 1.5 mPa·s, however, only small molecule components (i.e. non end‐group functionalized PEG) for the Biginelli reaction were tested. The small molecule Biginelli reaction was carried out in the viscous solution under identical VFD conditions as previous successful reactions (1 h, ambient conditions, 7000 rpm). After the reaction, no Biginelli products could be isolated for analysis. This test suggests that the drastic rate increase of the Biginelli reaction in the VFD takes place within the double helical, high‐shear vortices and not in the bulk‐flowing film. Furthermore, it appears that the critical double helical flow typically found in rpm regimes higher than around 6000 rpms is completely turned off in viscous fluids. Thus, viscosity appears to be a valuable means to tune and control the topological fluid flows within the VFD.

## 3 Conclusion

In conclusion, we demonstrate for the first time that Biginelli reactions can be greatly accelerated and carried out at ambient temperatures via high‐shear fluid flow. We also harnessed the versatility of the VFD to explore its potential in macromolecular chemistry. In particular, we investigated the efficiency of the Biginelli multicomponent reaction as a tool for post‐polymerization modifications. The VFD enabled the Biginelli reaction to occur with low catalyst loading, at ambient temperature, and in shorter reaction times when compared to traditional flask‐based Biginelli reactions. For small molecules, we observed full conversion to the Biginelli product. Additionally, we have highlighted the critical role that solution viscosity plays when using high‐shear fluid flows for Biginelli PPMs and that solutions below 1.5 mPa·s must be maintained for successful Biginelli reactions (both small molecule and PPMs). The viscosity of our reaction solutions emerged as a critical factor when using the VFD as a reaction vessel for reactions that involve materials that alter viscosity, and thus, future researchers must take this into account when considering reaction design in the VFD in batch mode. Currently, our team is exploring viscosity effects within the VFD under flow conditions.

## Conflict of Interest

The authors declare no conflict of interest.

## Supporting information



Supporting Information

## Data Availability

The data that support the findings of this study are available from the corresponding author upon reasonable request.
