# Supplementary material for: High‐Shear Enhancement of Biginelli Reactions in Macromolecular Viscous Media
Source: Macromol Rapid Commun. 2024 Sep 25;45(22):2400490. doi: 10.1002/marc.202400490 (PMC11583297; doi:10.1002/marc.202400490)
Supplement: Supplementary file 1 — Supporting Information [file MARC-45-2400490-s001.docx]

Supporting Information

High-shear enhancement of Biginelli reactions in macromolecular viscous media

Aaron Hung Bui, Naomi Beth Rowlands, Anne Dilpashani Fernando Pulle, Sam Andrés Gibbs Medina, Tullia Jade Rohrsheim, Bryan Tyler Tuten*

1. **Materials**

**Reagents & Solvents**

2-methoxyethanol (≥99.3%, Sigma-Aldrich), benzaldehyde (≥98%, Thermo Fisher Scientific), diethylether (99.9%, Thermo Fisher Scientific), ethanol (≥99.5%, Thermo Fisher Scientific), ethyl acetoacetate (≥98%, Merck), hydrochloric acid (32%, Thermo Fisher Scientific), poly(ethylene glycol) monomethyl ether/methoxypolyethylene glycol 350 (average mol wt 350, Sigma-Aldrich), poly(ethylene glycol) monomethyl ether (average Mn 550, Sigma-Aldrich), *tert*-butyl acetoacetate (98%, Sigma-Aldrich), toluene (≥99.5%, Thermo Fisher Scientific), urea (99.5%, Thermo Fisher Scientific)

1. **Characterization**

**Nuclear Magnetic Resonance spectroscopy**

^1^H spectra were recorded on a Bruker System 600 Ascend LH, equipped with a BBO-Probe (5 mm) with z-gradient (600.13 MHz). All measurements were carried out in deuterated solvents. The chemical shift (*δ)* is reported in parts per million (ppm) relative to tetramethylsilane (TMS). The *δ*-scale was calibrated to the respective solvent signal. *MestreNova 12.0* software was used to analyze spectra.

**Liquid chromatography-Mass spectrometry**

LC-MS measurements were performed on an UltiMate 3000 UHPLC System (Dionex, Sunnyvale, CA, USA) consisting of a pump (LPG 3400SZ), autosampler (WPS 3000TSL), and a temperature-controlled column compartment (TCC 3000). Separation was performed on a C18 HPLC column (Phenomenex Luna 5μm, 100 Å, 250 × 2.0 mm) operating at 40 °C. Water (containing 5 mmol L^-1^ ammonium acetate) and acetonitrile were used as eluents. A gradient of acetonitrile:water going from 5:95 to 100:0 (v/v) in 7 min at a flow rate of 0.40 mL·min^-1^ was applied. The flow was split in a 9:1 ratio, where 90 % of the eluent was directed through a DAD UV-detector (VWD 3400, Dionex) and 10 % was infused into the electrospray source. Spectra were recorded on an LTQ Orbitrap Elite mass spectrometer (Thermo Fisher Scientific, San Jose, CA, USA) equipped with a HESI II probe. The instrument was calibrated in the m/z range 74–1822 using premixed calibration solutions (Thermo Scientific). A constant spray voltage of 3.5 kV, a dimensionless sheath gas, and a dimensionless auxiliary gas flow rate of 5 and 2 were applied, respectively. The capillary temperature and was set to 300 °C, the S-lens RF level was set to 68, and the auxiliary gas heater temperature was set to 100 °C.

**Rheology**

Rheological experiments were conducted on the Anton Paar MCR302 Rheometer equipped with a Peltier temperature control (–20 to 200 °C). Low-viscosity measurements with a shear-rate sweep from 1 to 100 s^-1^ were conducted at room temperature using a concentric cylinder (CC27) and a cup stage measuring system for measuring liquids.

1. **Synthesis**

VFD experiments were conducted in a Vortex Fluidic Device with a hemispherical based tube (20 mm OD, 17.5 mm ID, length 19.3 cm).

**Biginelli Adduct (BA0)**

Ethyl acetoacetate (161 µL, 1.27 mmol, 1.52 eq), urea (50 mg, 0.83 mmol, 1 eq.) and benzaldehyde (85 µL, 0.83 mmol, 1 eq.) added to a VFD tube. 20 μL (one drop) of concentrated hydrochloric acid was added as well as 0.3 mL of ethanol. The reaction was carried out in the VFD at 7000 rpm and room temperature for 1 hr. After this, the solvent was evaporated using a stream of compressed air to yield a white solid product. Recovered: 30 mg. Yield: 14%.

^1^H NMR (600 MHz, DMSO-*d_6_*) δ 9.17 (s, 1 H), 7.72 (m, 1H), 7.33–7.22 (m, 5H), 5.14 (d, 1H), 3.98 (q, 2H), 2.24 (s, 3H), 1.09 (t, 3H).

HRMS (ESI): [C_14_H_16_N_2_O_3_+H]^+^ *m*/*z* (theoretical) 261.1234, *m*/*z* (experimental) 261.1235


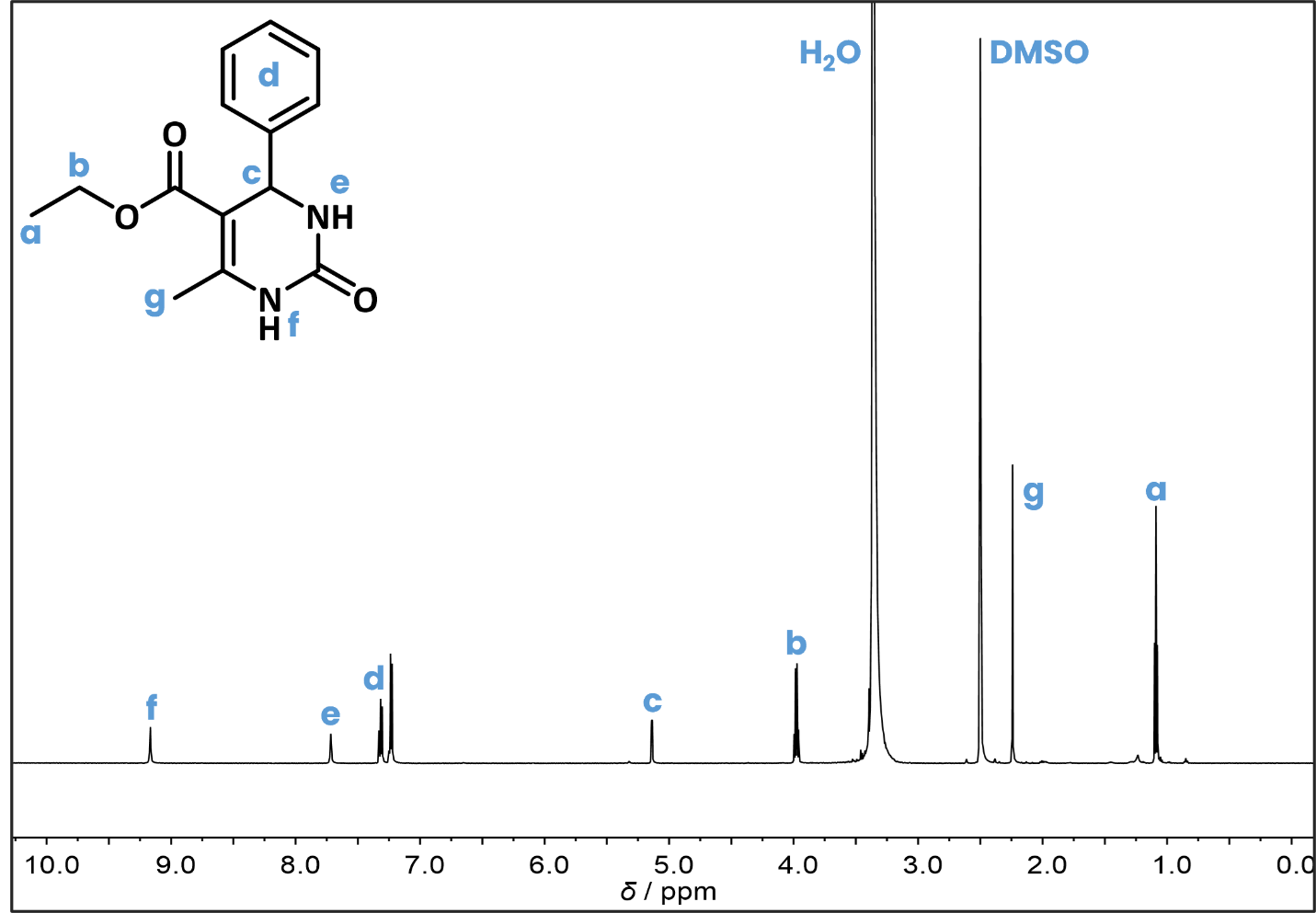


***Figure S1.*** *^1^H NMR spectra of* ***BA0****.*

**Acetoacetylation of 2-methoxy ethanol (AA1)**

2-Methoxy ethanol (518 µL, 6.57 mmol, 1 eq.) and tert-butyl acetoacetate (1090 µL, 6.57 mmol, 1 eq.) added to round-bottomed flask fitted with a Vigreux column. The reaction mixture was refluxed for 1 hr. Product was used in subsequent steps without further purification. 863 mg of product were recovered. Yield: 82%.

^1^H NMR (600 MHz, CDCl_3_): δ 4.19 (t, 2H), 3.51 (t, 2H), 3.40 (s, 2H), 3.28 (s, 3H), 2.17 (s, 3 H).

HRMS (ESI): [C_7_H_12_O_4_+H]^+^ *m*/*z* (theoretical) 161.0808, *m*/*z* (experimental) 161.0805.


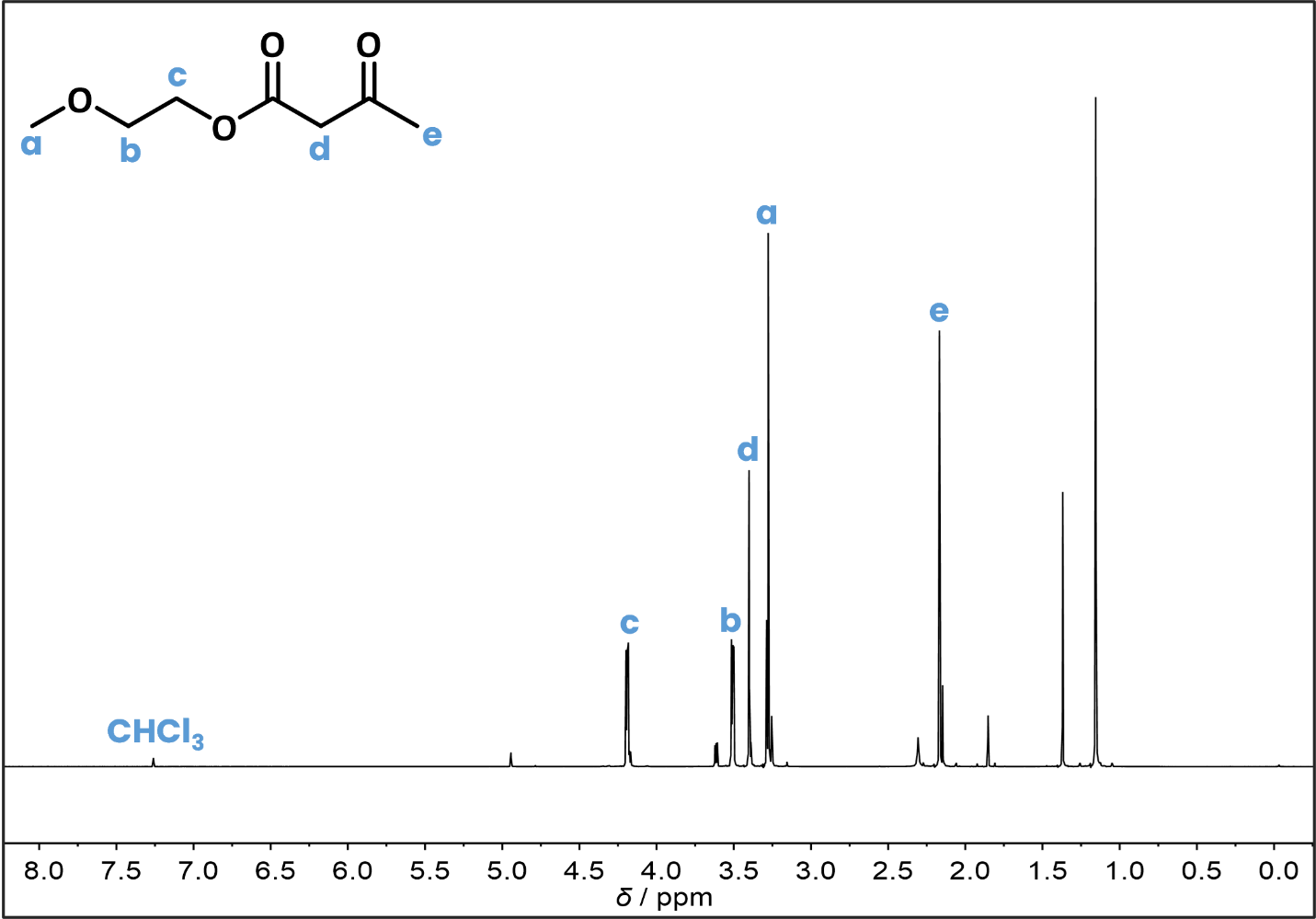


***Figure S2.*** *^1^H NMR spectrum of acetoacetylated 2-methoxy ethanol.*

**Acetoacetylated poly(ethylene glycol) monomethyl ether M_n_ 350 (AA2)**

500 mg of poly(ethylene glycol) monomethyl ether Mn 350, 0.5 mL of *tert*-butyl acetoacetate (large excess) and 1.5 mL of toluene added to round-bottomed flask fitted with a Vigreux column. The reaction mixture was refluxed for 30 min from the first occurrence of boiling. The solvent was removed via freeze-drying. 619 mg of product were recovered. Yield: 63%.

^1^H NMR (600 MHz, CDCl_3_) δ 4.29 (t, 2H), 3.70 (t, 2H), 3.65–3.63 (b), 3.54 (t, 2H), 3.48 (s, 2H), 3.37 (s, 3H), 2.27 (s, 3H).


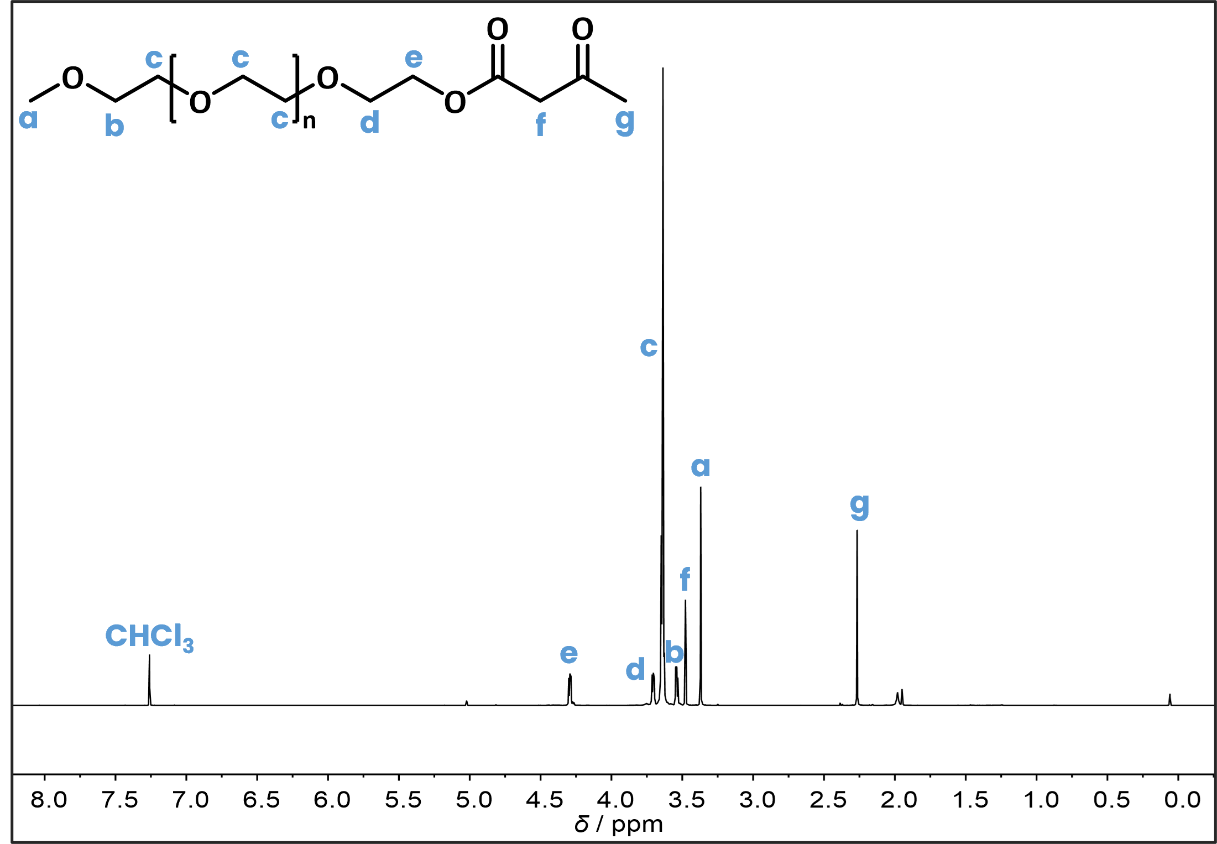


***Figure S3.*** *^1^H NMR spectrum of acetoacetylated poly(ethylene glycol) monomethyl ether Mn 350.*


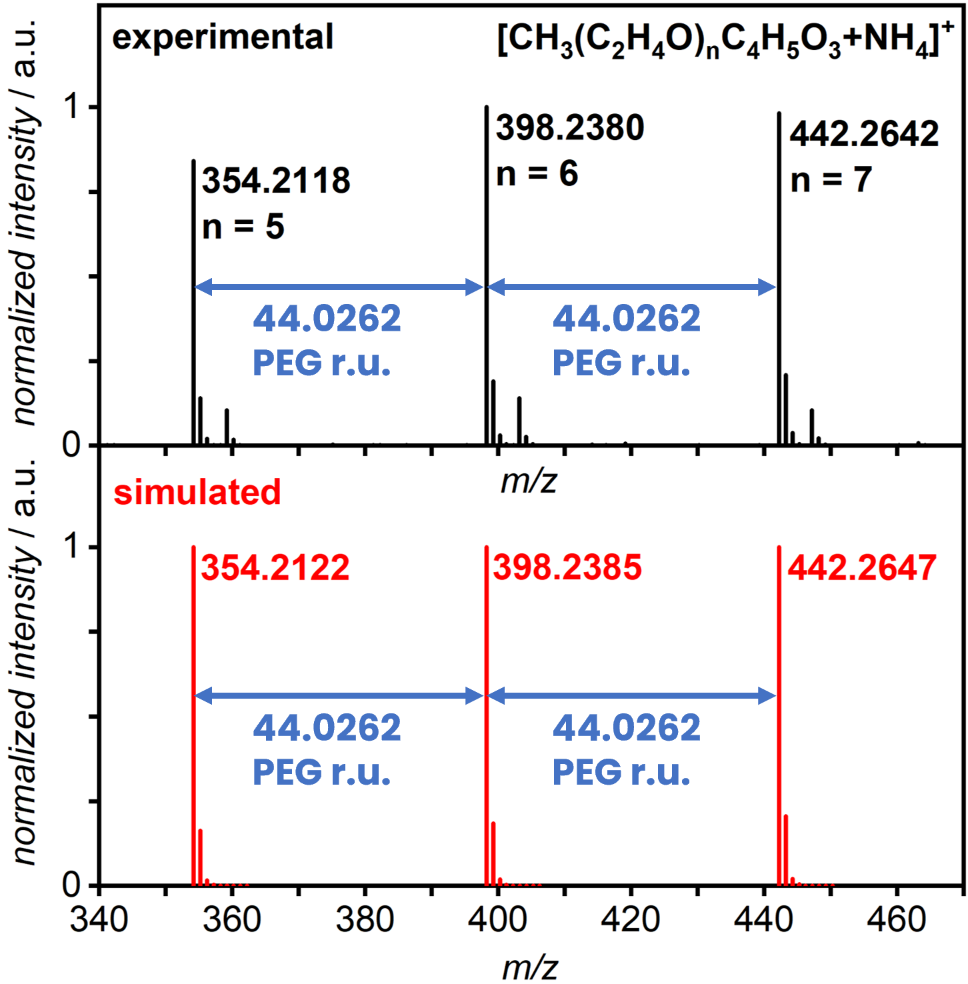


***Figure S4.*** *HRMS (ESI) of acetoacetylated poly(ethylene glycol) monomethyl ether Mn 350.*

**Acetoacetylated poly(ethylene glycol) monomethyl ether Mn 750 (AA3)**

500 mg of poly(ethylene glycol) monomethyl ether Mn 750, 0.5 mL of *tert*-butyl acetoacetate (large excess) and 1.5 mL of toluene added to round-bottomed flask fitted with a Vigreux column. The reaction mixture was refluxed for 30 min from the first occurrence of boiling. The solvent was removed through freeze-drying. 585 mg of product were recovered. Yield: 60%.

^1^H NMR (600 MHz, CDCl_3_) δ 4.29 (t, 2H), 3.70 (t, 2H), 3.65–3.63 (b), 3.54 (t, 2H), 3.48 (s, 2H), 3.37 (s, 3H), 2.27 (s, 3H).


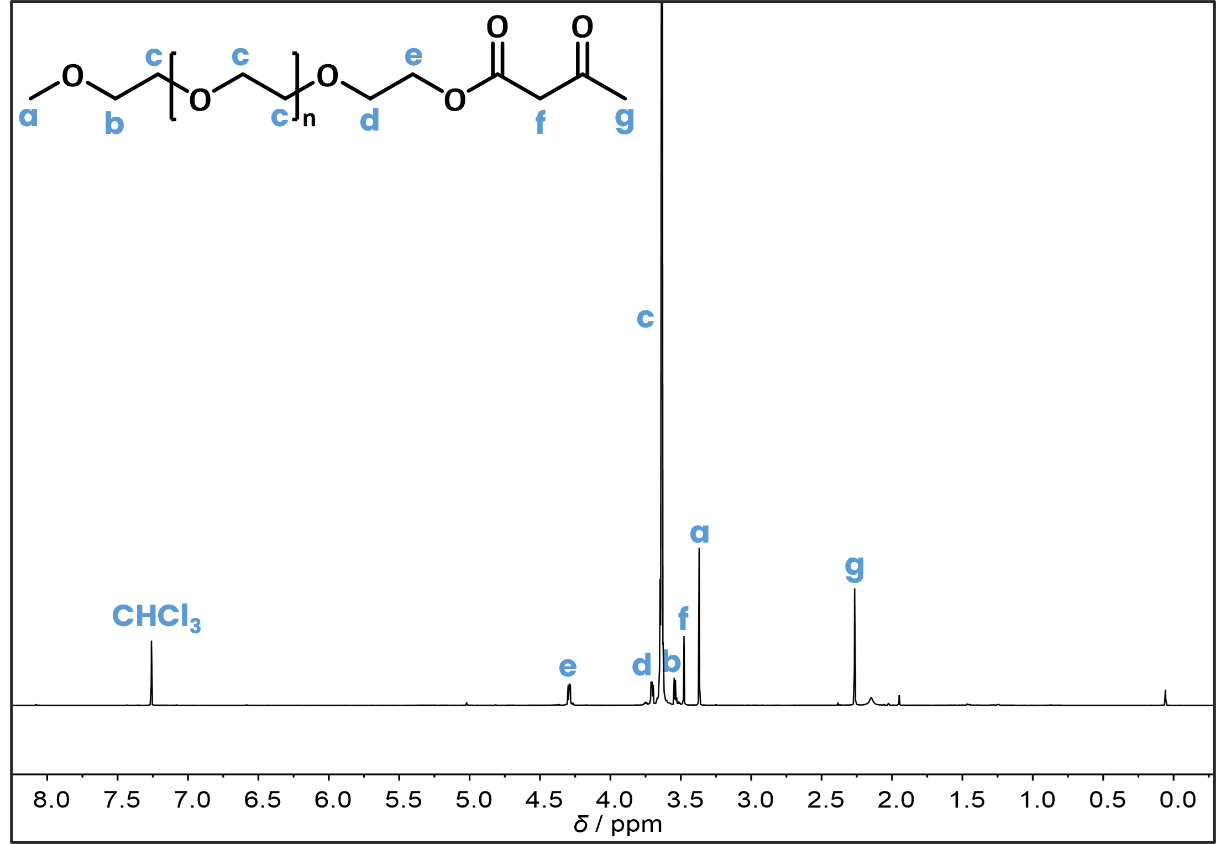


***Figure S5.*** *^1^H NMR spectrum of acetoacetylated poly(ethylene glycol) monomethyl ether Mn 750.*


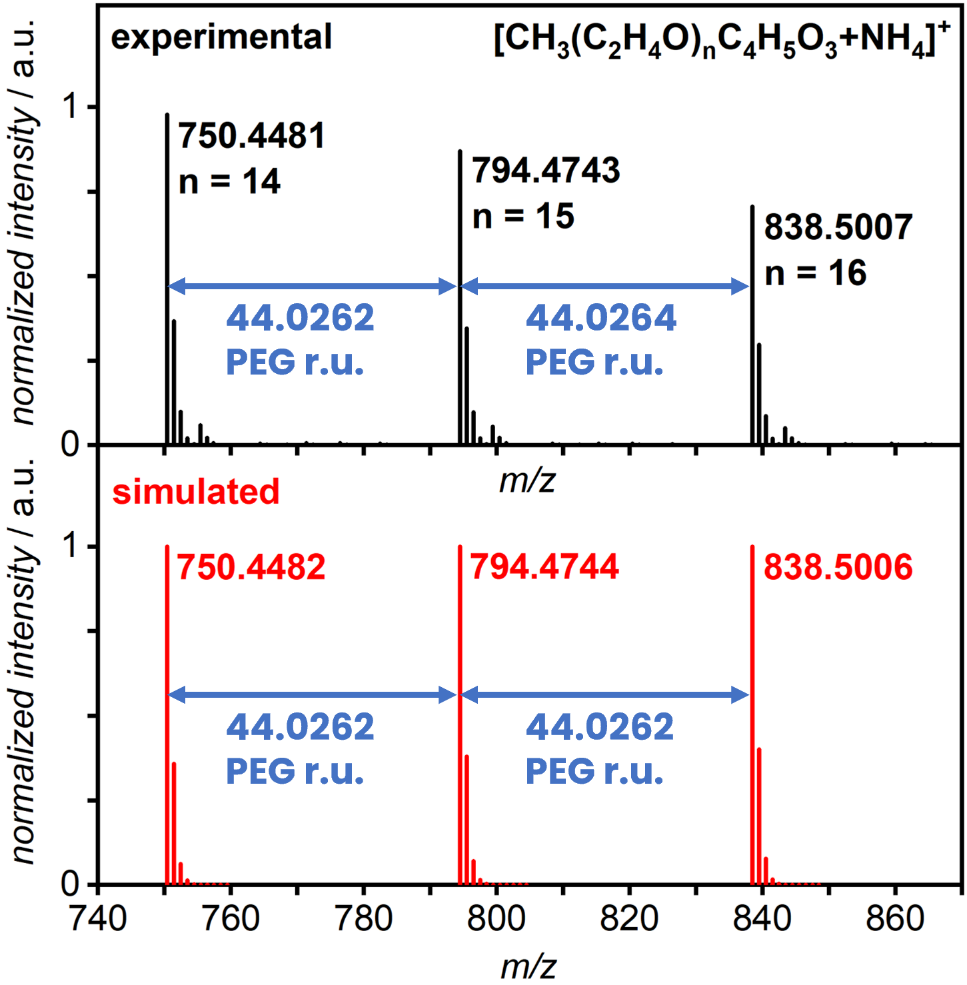


***Figure S6.*** *HRMS (ESI) of acetoacetylated poly(ethylene glycol) monomethyl ether Mn 750.*

**Biginelli reaction of AA1 (BA1)**

Stock solutions of urea in ethanol (20 mg/mL) and benzaldehyde in ethanol (20 mg/mL) were prepared. The following volumes used refer to the amount taken from the stock solutions.

To a VFD tube **AA1** (15 mg, 0.092 mmol, 1.52 eq.), urea (182 µL, 0.061 mmol, 1 eq.), benzaldehyde (318 µL, 0.061 mmol, 1 eq.) and 20 μL (one drop) of concentrated hydrochloric acid were added. The reaction was run in a VFD at 7000 rpm and room temperature for 1 hr. The product was dried in a vacuum oven overnight. 8 mg of product were recovered. Yield: 32%.

^1^H NMR (600 MHz, CDCl_3_) δ 8.04 (s, 1H), 7.35–7.25 (m, 5H), 5.71 (s, 1H), 5.43 (s, 1H), 4.20–4.13 (m, 2H), 3.52–3.44 (m, 2H), 3.31 (s, 3H), 2.35 (s, 3H).

HRMS (ESI): [C_15_H_18_N_2_O_4_+H]^+^ *m*/*z* (theoretical) 291.1339, *m*/*z* (experimental) 291.1338.


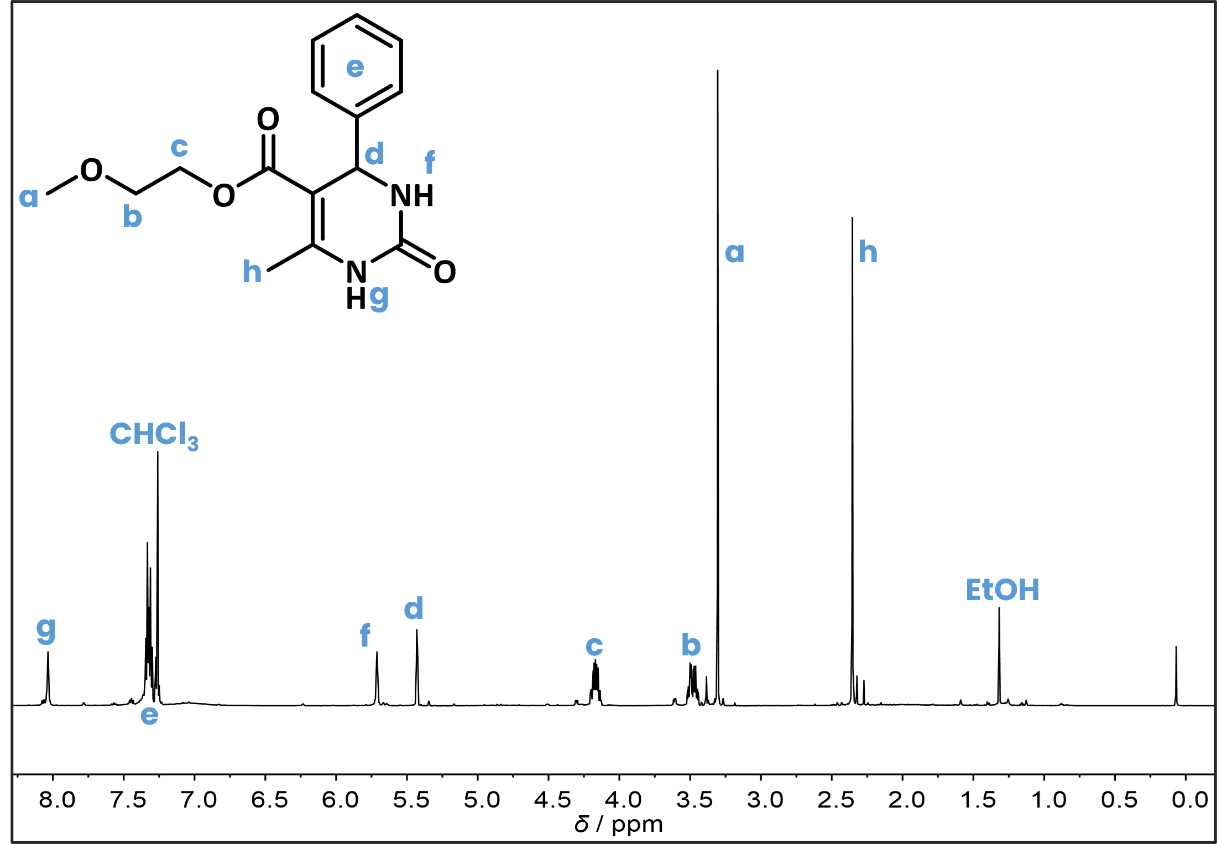


***Figure S7.*** *^1^H NMR spectrum of* ***BA1****.*

**Biginelli reaction of AA2 (BA2)**

Stock solutions of urea in ethanol (20 mg/mL) and benzaldehyde in ethanol (20 mg/mL) were prepared. The following volumes used refer to the amount taken from the stock solutions.

To a VFD tube **AA2** (32 mg, 0.092 mmol, 1.52 eq.), urea (182 µL, 0.061 mmol, 1 eq.), benzaldehyde (318 µL, 0.061 mmol, 1 eq.) and 20 μL (one drop) of concentrated hydrochloric acid were added. The reaction was run in a VFD at 7000 rpm and room temperature for 6 hrs. The product was dried in a vacuum oven overnight. 26 mg of product were recovered. Yield: 62%.

^1^H NMR (600 MHz, CDCl_3_) δ 8.05 (s, 1H), 7.35–7.23 (m, 5H), 5.93 (s, 1H), 5.42 (s, 1H), 4.29 (t, 2H), 3.72–3.69 (m, 2H), 3.65–3.59 (b) 3.59–3.56 (m, 2H), 3.37 (s, 3H), 2.36 (s, 3H).


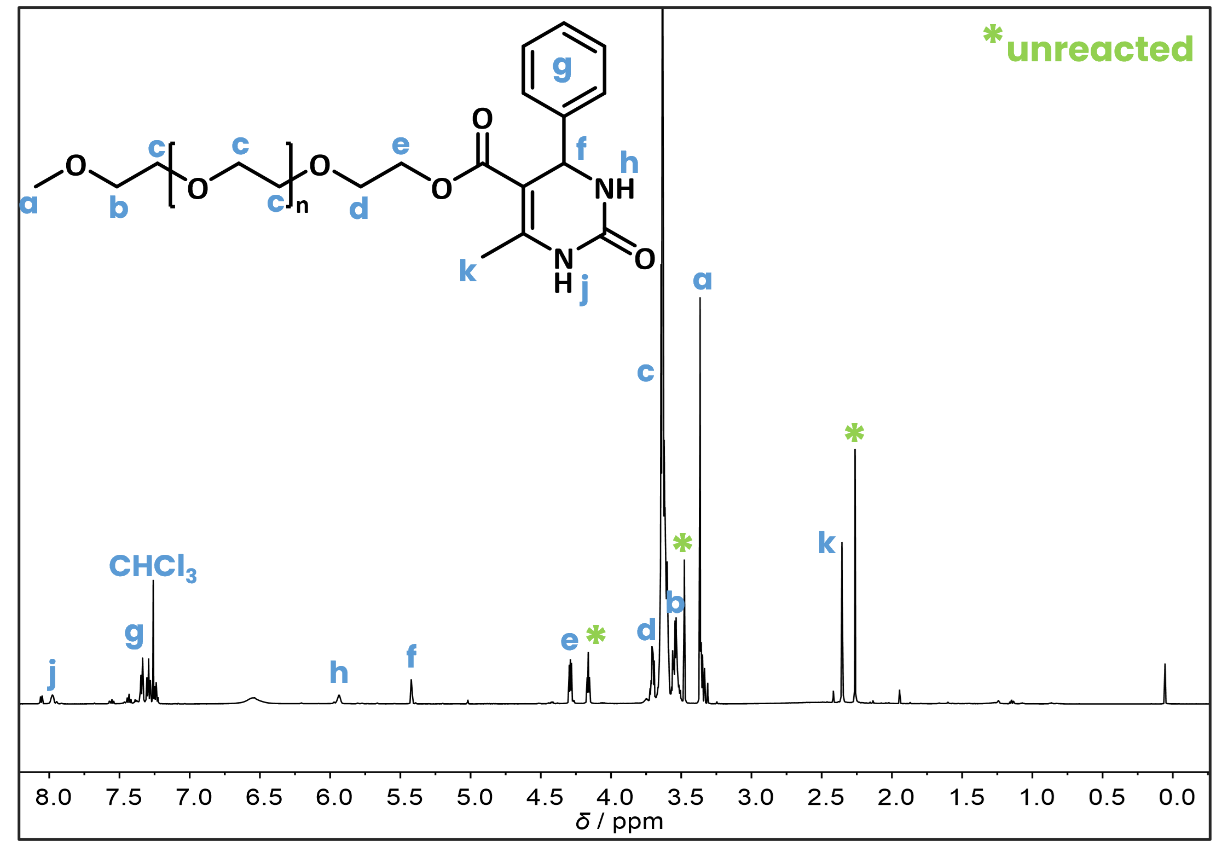


***Figure S8.*** *^1^H NMR spectrum of* ***BA2****.*

4
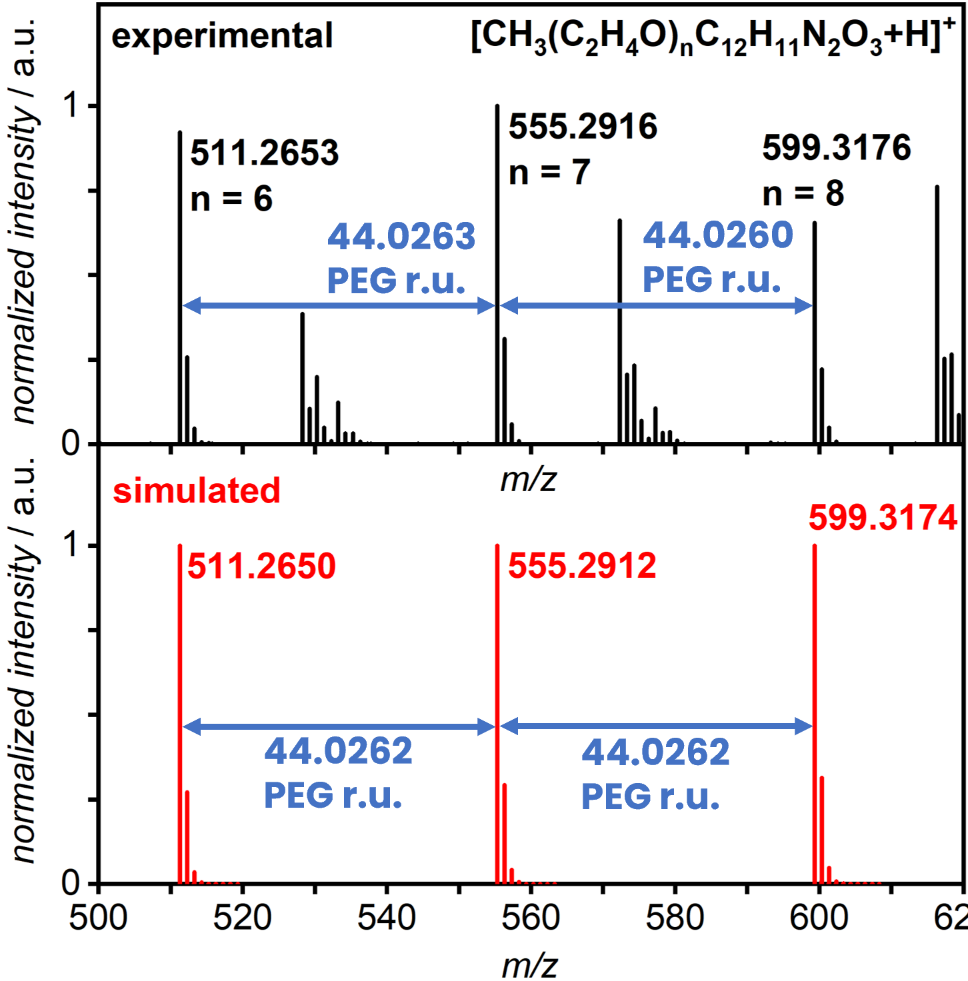


***Figure S9.*** *HRMS (ESI) of* ***BA2****.*

**Biginelli reaction of AA3 (BA3)**

Stock solutions of urea in ethanol (20 mg/mL) and benzaldehyde in ethanol (20 mg/mL) were prepared. The following volumes used refer to the amount taken from the stock solutions.

To a VFD tube **AA3** (69 mg, 0.092 mmol, 1.52 eq.), urea (182 µL, 0.061 mmol, 1 eq.), benzaldehyde (318 µL, 0.061 mmol, 1 eq.) and 20 μL (one drop) of concentrated hydrochloric acid were added. The reaction was run in a VFD at 7000 rpm and room temperature for 6 hrs. The reaction mixture was precipitated into cold n-pentane and centrifuged to decant the solvent. The product was dried in a vacuum oven overnight. 60 mg of product were recovered.

**Biginelli reaction in Viscous PEG750**

Stock solutions of urea in ethanol (20 mg/mL) and benzaldehyde in ethanol (20 mg/mL) were prepared.

To a VFD tube Poly(ethylene glycol) monomethyl ether with average Mn 750 (69 mg, 0.092mmol, 1.52 eq.), urea stock solution (182 uL, 0.061 mmol, 1 eq.), benzaldehyde stock solution (318 uL, 0.061mmol, 1eq.) and ethyl acetoacetate (12mg, 0.092, 1.52 eq.) were swirled until the Poly(ethylene glycol) monomethyl ether had dissolved into solution. 20 μL (one drop) of hydrochloric acid 32% was added to the solution and a modified rubber stopper was used to seal the VFD tube. The VFD due was run at 7000 rpm at a 45° incline for 1 hour at room temperature. After precipitation into cold n-pentane, no product could be recovered.


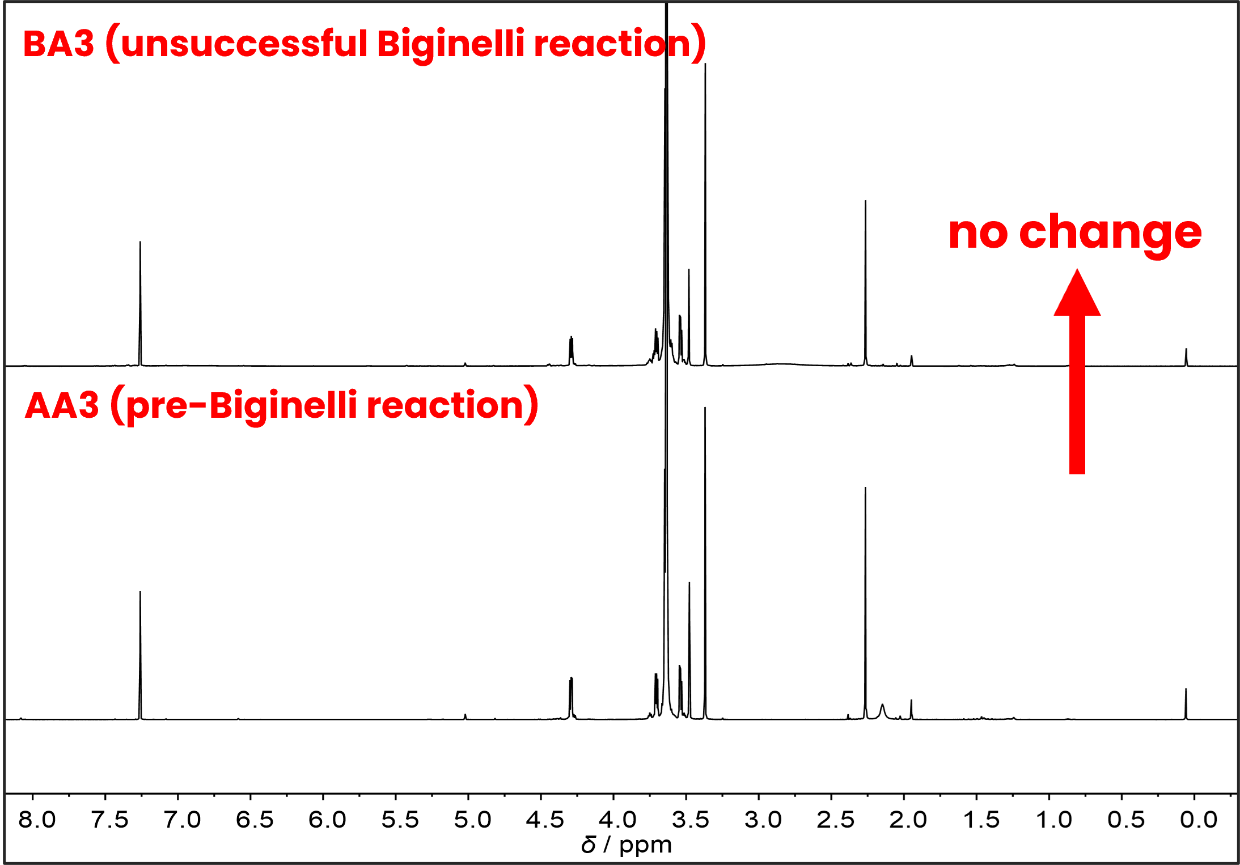


***Figure S10.*** *^1^H NMR spectrum of* ***BA3****.*


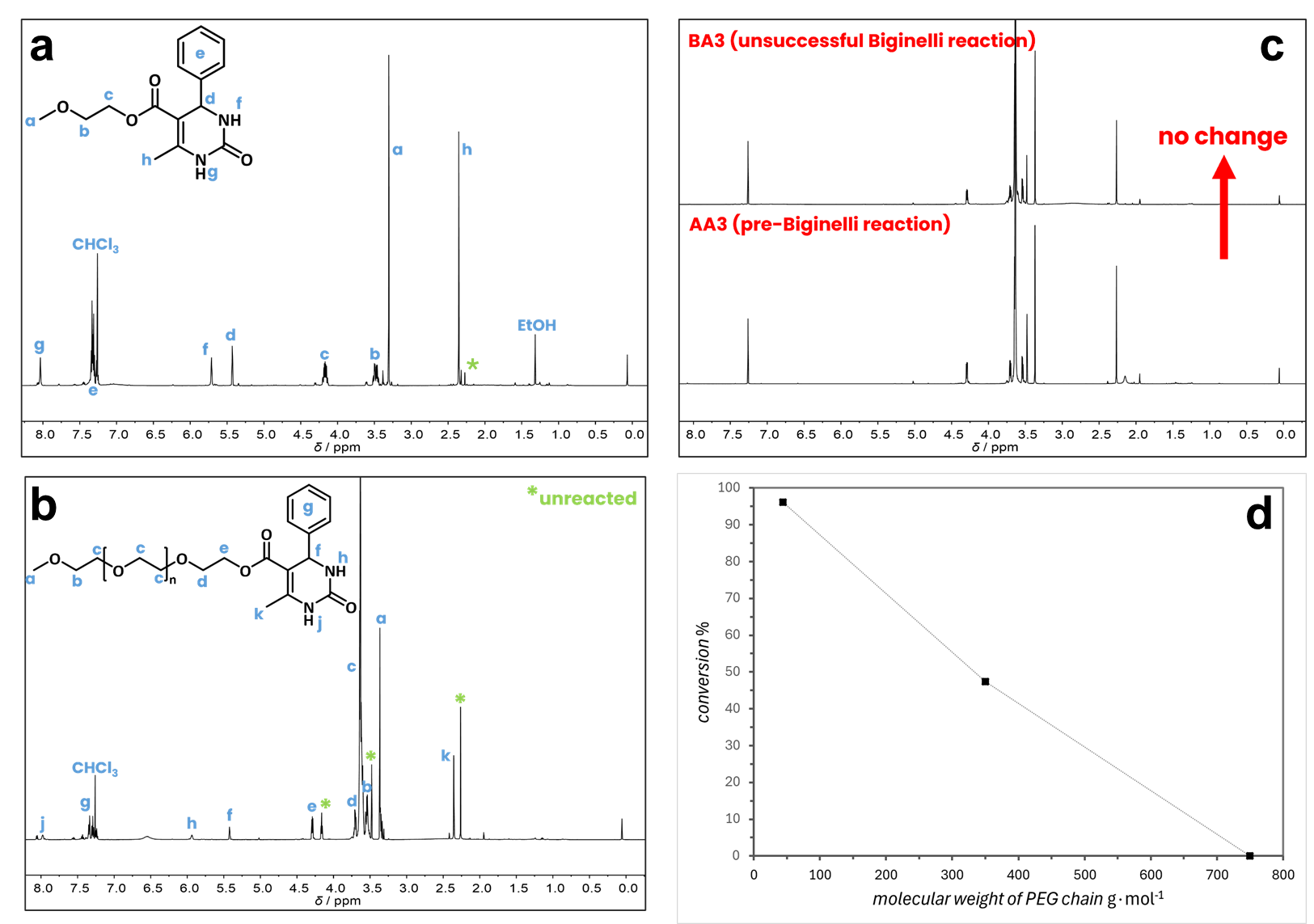


**Figure S 11** Overview of PEG chain length effects on yield. **a**) “PEG-0” indicating 96.2% yield based off integrals h and * (3.00 to 0.12). **b**) PEG-350 indicating 48.0% yield based off integrals k and k* (3.00 to 3.24) and 47.4% yield based off e and e* (2.12 to 2.35). **c**) PEG-750 indicating no conversion to the Biginelli product. **d**) Plot of yield as a function of PEG molecular weight.
